# Sex Differences in Genetic and Environmental Influences on Adolescent Depressive Symptoms: A Meta-Analytic Review

**DOI:** 10.1155/2015/476238

**Published:** 2015-11-15

**Authors:** Jie Chen, Jing Yu

**Affiliations:** ^1^Key Laboratory of Mental Health, Institute of Psychology, Chinese Academy of Sciences, Beijing 100101, China; ^2^Department of Psychology, University of Maryland, Baltimore County, MD 21250, USA

## Abstract

Although sex difference in the mean level of depressive symptoms has been well established, the sex difference in genetic and environmental influences on adolescent depressive symptoms is unclear. The current study conducted a meta-analysis of twin studies on sex differences in self- and parent-reported adolescent depressive symptoms. For self-reports, genetic factors influenced adolescent depressive symptoms equally for boys and girls, accounting for 46% of variation, but shared environmental factors had stronger impacts on adolescent girls' versus boys' depressive symptoms (13% versus 1% of the variance). For parent-reports, genetic, shared, and nonshared environmental factors influenced adolescent depressive symptoms equally, with separate estimates of 34%, 35%, and 31%. The implications of sex difference in genetic and environmental etiologies of depressive symptoms are discussed.

## 1. Introduction

Research shows that sex differences in depression emerge in adolescence and continue into adulthood [1, 2]. In childhood, girls are no more depressed than boys, but more girls than boys start to exhibit depressive symptoms [3, 4] and clinical depression [5] at approximately the age of 13. It has been hypothesized that the etiology of sex difference in depression rests upon differential influences of genetic and environmental factors between boys and girls [6, 7]. This differential influence could be either qualitative (i.e., there are some sex-specific genetic and/or environmental contributors to adolescent depression) or quantitative (i.e., the same genetic and environmental factors contribute to boys' and girls' depression, but these contributors influence the two sexes unequally). This study adopts a meta-analytic review to address two questions: Which kind of etiological effect, qualitative or quantitative, accounts for the sex differences in adolescent depression? And, if it is the latter, how and to what extent do the same genetic and environmental factors influence the two sexes differently?

We are unaware of any meta-analytic review that has focused specifically on sex differences in genetic and environmental etiologies of adolescent depression. One comprehensive narrative review concluded that there were no qualitative sex differences in genetic and environmental etiologies of childhood and adolescent depression, but the findings of quantitative sex differences varied across studies [8]. For example, two studies found stronger genetic effects on adolescent boys' than on adolescent girls' self-reported depressive symptoms, but there was no significant difference across sex for parent-rated data [9, 10]. Three studies reported larger genetic influences on self-reported depression symptoms for female than for male adolescents [11–13]. Scourfield et al. [14] found that girls showed greater genetic influence than boys for parent-reported data but found no significant difference for self-report. In addition, three studies reported no sex differences in the magnitudes of genetic and environmental influences on both self- and parent-reported adolescent depression symptoms [15–17]. Given the inconsistent findings among previous research, a meta-analytic review is needed to quantitatively assess the genetic and environmental influences on adolescent depression in boys and girls.

One of the criticisms of meta-analysis is the apples and oranges argument: the meta-analysis is analogous to taking apples and organs and averaging such measures as their weights, sizes, flavors, and shelf lives [18]. This argument criticizes that meta-analysis simply summarizes results from studies that vary notably in their operationalization and measures of study variables and that employ very different samples. Simply combining the results of methodologically heterogeneous studies can lead to misleading conclusion. To overcome the heterogeneity issue, two approaches can be used. One is to select the more methodologically homogeneous primary studies, and the other is to investigate the source of heterogeneity and examine the moderators of the heterogeneous findings [19].

With regard to the topic of the current study, several moderating variables should be taken into account. The first is the age of study samples. Previous studies have demonstrated that the estimates of genetic influences on depression increased from childhood to adolescence [9, 14]. The second lies in the operationalization of depression as diagnosed depressive disorder or questionnaire-measured depressive symptoms. Studies showed that genetic influences on depression were significantly larger when assessed via questionnaire as opposed to diagnostic interview [15]. The third related to informants (parents versus adolescents themselves). The shared environmental effects were found larger in parents-reported data as opposed to self-reported data [16]. Investigating these moderator effects is definitely interesting, but the amount of included studies must be large enough to conduct a thorough analysis. Furthermore, the confounding and interaction among moderators are difficult to solve without enough large amount of included studies [20]. For example, there are still heterogeneities in age and informants in questionnaire-based studies, while heterogeneities in informants and operationalization also exist in childhood studies.

Therefore, in the current study, we adopted the first approach: select more homogeneous studies in meta-analytic review. Specifically, we selected twin studies that use child-reported or parent-reported questionnaire-assessed depressive symptoms in adolescents. The specific questions we want to answer are whether there are qualitative or quantitative sex differences in genetic and environmental etiologies of adolescent depressive symptoms? If there are quantitative sex differences, what are the quantities of genetic and environmental effects on adolescent depressive symptoms in boys and girls? We conduct the meta-analyses separately for self- and parent-reported data.

## 2. Method

### 2.1. Search Strategy

To identify relevant journal articles, we first searched the PsycINFO and Medline databases (in July 2015) using the following terms: twins, twin study, depression, and depressive symptoms. Only articles whose abstracts clearly reported the use of a child or adolescent sample were examined. We also reviewed the references reported in the selected papers in search of additional relevant articles. The strategy yielded a total of 46 studies, four of which were excluded because they were reviews or meta-analyses.

The 42 empirical studies were carefully reviewed to determine whether they met the following inclusion criteria. First, given that the estimates of genetic and environmental effects vary across age (childhood versus adolescence), assessment methods (diagnostic interview versus questionnaire), and informants sources (children/adolescents themselves, parent, peer, and teacher) and to reduce heterogeneity in the included studies, the current meta-analysis mainly focused on twin studies with survey-based self- or parent-reports of depressive symptoms in adolescents (aged 11 to 19 years) (15 studies were excluded). Second, because the current study aimed to examine both qualitative and quantitative sex differences in genetic and environmental influences on adolescent depressive symptoms, the selected studies had to report effect sizes of intraclass correlations or Pearson correlations for the following five groups of twin pairs: male monozygotic (MZM) twins, male dizygotic (DZM) twins, female monozygotic (MZF) twins, female dizygotic twins (DZF), and opposite-sex dizygotic (DZOS) twins (8 studies were excluded). Third, only studies with Western adolescent populations were selected for the current meta-analysis (2 studies were excluded). Finally, each study included in the current meta-analysis was based on independent samples; in the case where several studies were published from the same dataset, we selected the one with the largest sample size and/or the study that employed univariate models (12 studies were excluded). The application of the aforementioned inclusion criteria resulted in the identification of 5 twin studies on adolescent depressive symptoms (see Table 1).

### 2.2. Data Analysis

As the current study aimed to clarify both qualitative and quantitative sex differences in genetic and environmental influences on adolescent depressive symptoms, the intraclass or Pearson product-moment correlations of five twin groups (i.e., MZM, MZF, DZM, DZF, and DZOS) were used as effect sizes and were analyzed in the model-fitting program Mx [21].

**Figure 1:**
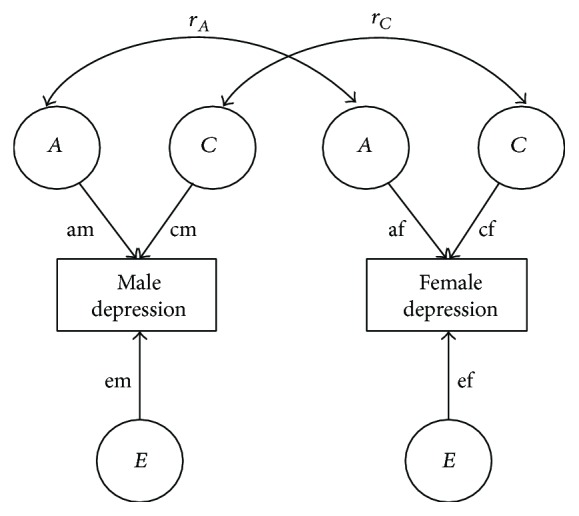
Sex-limitation model for adolescent depressive symptoms. The magnitude of additive genetic (*A*), shared environmental (*C*), and nonshared environmental (*E*) influences may differ for males and females (am ≠ af, cm ≠ cf, and em ≠ ef), and/or the genetic (*r*_*A*_) or shared environmental (*r*_*C*_) correlation among opposite-sex twins may fall below the expected genetic (.50) and shared environmental (1.00) correlations for same-sex dizygotic twins.

To examine both qualitative and quantitative sex differences, it is necessary to first fit a full sex-limitation model and then two nested submodels, which progressively model fewer parameters (Figure 1). In* the full sex-limitation model*, additive genetic (*A*), shared (*C*), and nonshared environmental (*E*) parameters were allowed to differ between males and females, assuming that the magnitudes of influence of *A*, *C*, and *E* on depressive symptoms may vary in males and females. For DZOS twins, the correlations for *A* factors (*r*_*A*_) and *C* factors (*r*_*C*_) were estimated freely. Because a model estimating both *r*_*A*_ and *r*_*C*_ simultaneously is not identifiable, the two correlations were estimated separately in two nonnested models. The fits of these two nonnested models were compared with the Akaike information criterion (AIC) and the model with the smaller AIC was selected as the best fitting model. Then, data were fitted into the first nested submodel,* the common effect model*, which constrained DZOS twins' *r*_*A*_ to 0.5 or *r*_*C*_ to 1.0 but allowed *A*, *C*, and *E* parameters for males and females to differ. The significance of difference in fits between the common effect model and the full sex-limitation model was tested to examine qualitative sex differences. The second nested submodel is* the scalar model*, which constrains DZOS twins' *r*_*A*_ to 0.5 or *r*_*C*_ to 1.0 and allows *A*, *C*, and *E* parameters for males and females to be equal. The significance of difference in fits between the scalar model and the common effect model tested quantitative sex differences.

## 3. Results

The model-fitting results and parameter estimates for the twin data included in the meta-analysis are presented in Table 2. For self-reported data, the fits of the two nonnested full sex-limitation models (full *r*_*A*_ and *r*_*C*_) were very similar; therefore, we selected the full *r*_*A*_ model as the baseline model. When the freely estimated *r*_*A*_ (0.21) was set to 0.5 (the common effect model), there was no significant change in chi-square, Δ*χ* ^2^(1) = 2.41, *p* > .05. However, further allowing the genetic and environmental estimates (path coefficients for *A*, *C*, and *E* factors) to be equal across sexes (the scalar model) resulted in a significant change in chi-square, Δ*χ* ^2^(3) = 15.91, *p* < .01. These findings suggest a significant quantitative sex difference without a qualitative sex difference: although adolescent boys and girls share the same genetic and environmental factors, the extents to which they are influenced by these factors differ.

To further examine which factor (*A*, *C*, or *E*) could account for this unequal quantitative influence, three submodels of the common effect model (set am = af, cm = cf, and em = ef; see Figure 1 for details) were tested, and the model fits were compared with that of the common effect model. The results showed no significant chi-square change when am and af were set to be equal, Δ*χ* ^2^(1) = 2.49, *p* > .1, but significant chi-square changes when cm and cf or em and ef were set to be equal, respectively, Δ*χ* ^2^(1) = 5.85, *p* < .01; Δ*χ* ^2^(1) = 5.85 (1), *p* < .01. Thus, the quantitative difference in environmental influences, rather than genetic influences, is likely to be the source of the phenotypic sex difference. Considering goodness of fit and parsimoniousness, the model with am = af was considered to be the best fitting model. In this model, shared environmental influences were higher in girls than in boys, whereas nonshared environmental estimates were higher in boys than in girls.

For parent-reported data, the full *r*_*A*_ model was also selected as the baseline model, as the fits of the two nonnested full sex-limitation models (full *r*_*A*_ and *r*_*C*_) were very similar. When the freely estimated *r*_*A*_ (0.48) was set to 0.5 (common effect model), there was no significant change in chi-square, Δ*χ* ^2^(1) = 0.07, *p* > .2. Additionally, further allowing the genetic and environmental estimates to be equal across sexes (scalar model) resulted in a nonsignificant change in chi-square, Δ*χ* ^2^(3) = 3.72, *p* > .2. These findings suggest that, for the parent-reported data, there was neither a quantitative nor a qualitative influence on adolescent boys and girls: adolescent boys and girls shared the same genetic and environmental risk factors, and these factors influenced their depressive symptoms equally.

## 4. Discussion

Findings from prior studies regarding sex differences in genetic and environmental influences on adolescent depressive symptoms are inconsistent. We addressed this concern by conducting a meta-analysis of existing twin studies of adolescent depressive symptoms. We found no qualitative sex differences in genetic and environmental etiologies of adolescent depressive symptoms.

We found evidence for quantitative sex differences in self-reported data. The genetic factors contributed equally to depressive symptoms across sexes (explaining 46% of the variation), but shared environmental factors contributed a larger proportion of the total variance to adolescent girls' depressive symptoms (explaining 13% of the variation) than to adolescent boys' depressive symptoms (explaining 1% of the variation). Although we found the larger nonshared environmental estimates (53% in boys versus 41% in girls), the source of these sex differences is unclear as the nonshared environmental estimates include both true unique environmental effects and measurement errors.

The findings of the current study suggested that self-reported adolescent depressive symptoms are moderately influenced by genetic and nonshared environmental factors, which is consistent with findings of prior narrative literature reviews [8]. Furthermore, inconsistent with prior indication that shared environmental effects were negligible [22], we found modest shared environmental influences on depressive symptoms in adolescent girls. This finding is consistent with some empirical studies that have examined the influences of specific shared environmental factors on adolescent depressive symptoms. For example, family discord was found to exert greater impact on depressive symptoms for girls than for boys [23]. Parental distress and marital discord have been associated with depressive symptoms in adolescent girls but not in adolescent boys [24]. There is also evidence of a significant correlation between maternal depressive symptoms and adolescents' depressive symptoms for girls but not boys, which has mainly been explained by social disadvantage, marital discord, and family adversity [25, 26].

As twin studies of parent-reported adolescent depressive symptoms were relatively rare, only two studies were included in the current meta-analysis. We found no sex differences in genetic, shared, and nonshared environmental influences on parent-reported depressive symptoms, with estimates of 34%, 35%, and 31%, respectively. The larger estimates of shared environmental influences on parent-reported depression are likely due to a shared informant effect, as one parent provided reports on both twins. With the relatively smaller sample size in parent-reported data, our findings may be explained with caution regarding sex differences.

Several limitations should be mentioned. First, our findings were limited to adolescent sample and continuous depressive symptoms and may differ from those in research on adult depressive disorder. In fact, studies in adult twins, especially those with large-size samples, seemed to consistently report that major depression was appreciably more heritable in women than in men. For example, in one study with 2685 Australian twin pairs, the heritability of major depression was estimated at 44% in women and 24% in men [27]. Based on 2974 twin pairs from the population-based Virginia Twin Registry, Kendler et al. [28] found that the heritability of major depression was estimated at 40% in women and 31% in men. Using 15,493 twin pairs form the national Swedish Twin Registry, Kendler et al. [29] showed that the heritability of major depression was significantly higher in women (42%) than men (29%). Furthermore, studies of depressive symptoms in elderly adults also found higher heritability in women than in men [30, 31]. Future studies need to explore the possible mechanism underlying this developmental difference.

Second, our meta-analysis has not included research on adolescent anxious/depressive symptoms. However, these researches found similar findings to us. Specifically, two studies using the large adolescent sample from the Netherlands Twin Register (NTR) reported no quantitative and qualitative sex difference in genetic influences on adolescent anxious/depressive symptoms [32, 33]. Third, our meta-analysis selected studies in Western adolescents; as studies in non-Western sample start growing [34], future studies can investigate the cultural difference.

Given the limitation mentioned above, findings from the current study have potential implications for future investigating the etiology and intervention of adolescent depressive symptoms. First, we found that the same genetic factors equally influence adolescent girls' and boys' depressive symptoms, indicating that the specific or candidate genes related to adolescent depressive symptoms might operate in a transgender mechanism. Second, we found that shared environmental risk factors exerted stranger effects on depressive symptoms in adolescent girls as opposed to adolescent boys. This suggests the potential benefit of focusing on intervention targets within the shared environmental system (e.g., family poverty, parental psychopathology, and marital discord) to help prevent the onset of depression among adolescent girls.

## 5. Conclusions

We conducted a meta-analysis of sex differences in genetic and environmental influences on adolescent depressive symptoms. There were no qualitative sex differences in genetic and environmental etiologies of adolescent depressive symptoms. We found evidence for quantitative sex differences. The same genetic factors affected adolescent depressive symptoms equally, and shared environmental risk factors exerted stranger effects in adolescent girls' depressive symptoms. Results of this study indicate the potential benefit of focusing on intervention targets within the shared environmental system to help prevent the onset of depression among adolescent girls.

## Figures and Tables

**Table 1:** Studies included in the meta-analysis.

Sample and Study	Instrument	Informant	Age	Twins	*N*	Effect size
Cardiff Study (CaStANET) [9]	MFQ	Mother	8–17	MZ_M MZ_F DZ_M DZ_F DZ_OS	262 323 178 231 420	.71 .79 .64 .69 .59
	MFQ	Child	11–17	MZ_M MZ_F DZ_M DZ_F DZ_OS	153 209 111 156 251	.54 .61 .32 .45 .23

Registry for Child Twins [10]	CDI	Child	12–16	MZ_M MZ_F DZ_M DZ_F DZ_OS	50 64 44 44 42	.63 .61 .22 .54 .10

Finn Twin [16]	MPNI	Mother	12	MZ_M MZ_F DZ_M DZ_F DZ_OS	213 245 242 223 443	.60 .60 .40 .38 .44
	CDI	Child	12	MZ_M MZ_F DZ_M DZ_F DZ_OS	89 108 84 73 145	.42 .54 .25 .24 .16

Add Health [12]	CES-D	Child	11–19	MZ_M MZ_F DZ_M DZ_F DZ_OS	141 141 131 114 197	.35 .54 .35 .21 .16

G1219 [17]	MFQ	Child	12–19	MZ_M MZ_F DZ_M DZ_F DZ_OS	168 199 138 190 463	.52 .57 .33 .49 .34

MRQ: Mood and Feelings Questionnaire.

MPNI: Multidimensional Peer Nomination Inventory; CDI: Children Depression Inventory.

**Table 2:** Parameter estimates and fit indices for available twin data included in meta-analysis.

	df	*χ* ^2^	AIC	Male			Female			Opposite-sex twins	
				*A*	*C*	*E*	*A*	*C*	*E*	*r*_*A*_	*r*_*C*_
Child-reported											
Full *r*_*A*_	68	35.28	−100.72	.33 (.15, .52)	.14 (.00, .30)	.52 (.47, .58)	.36 (.21, .51)	.22 (.08, .35)	.43 (.38, .47)	.21 (.00, .05)	1.0
Full *r*_*C*_	68	35.28	−100.71	.33 (.15, .52)	.14 (.00, .29)	.52 (.46, .58)	.36 (.21, .51)	.22 (.08, .35)	.43 (.38, .47)	.50	.43 (−.08, 1.0)
Common	69	37.70	−100.30	.48 (.39, .54)	.01 (.00, .07)	.51 (.46, .57)	.35 (.19, .51)	.23 (.08, .36)	.43 (.38, .48)	.50	1.0
Scalar	72	53.61	−90.39	.45 (.36, .56)	.07 (.00, .15)	.47 (.43, .51)	.45 (.36, .56)	.07 (.00, .15)	.47 (.43, .51)	.50	1.0
am = af	**70**	**40.19**	**−99.81**	**.46 (.35, .53)**	**.01 (.00, .10)**	.**53 (.47, .58)**	.**46 (.35, .53)**	.**13 (.05, .23)**	**.41 (.37, .46)**	**.50**	**1.0**
cm = cf	70	43.55	−96.45	.39 (.27, .50)	.08 (.00, .16)	.53 (.47, .59)	.50 (.39, .60)	.08 (.00, .16)	.42 (.38, .47)	.50	1.0
em = ef	70	43.23	−96.77	.51 (.44, .56)	.02 (.00, .07)	.47 (.43, .51)	.27 (.13, .42)	.27 (.12, .39)	.47 (.43, .51)	.50	1.0
Parent-reported											
Full *r*_*A*_	23	91.00	45.00	.31 (.17, .45)	.34 (.23, .47)	.34 (.30, .39)	.34 (.21, .46)	.37 (.26, .48)	.29 (.26, .33)	.48 (.24, .50)	1.0
Full *r*_*C*_	23	91.00	45.00	.31 (.17, .44)	.34 (.23, .47)	.34 (.30, .39)	.34 (.21, .46)	.37 (.26, .48)	.29 (.26, .33)	.50	.98 (.83, 1.0)
Common	24	91.04	43.04	.33 (.17, .45)	.34 (.23, .46)	.34 (.30, .39)	.35 (.22, .46)	.36 (.26, .48)	.29 (.26, .33)	.50	1.0
Scalar	**27**	**94.76**	**40.76**	**.34 (.26, .42)**	**.35 (.28, .41)**	**.31 (.29, .34)**	**.34 (.27, .41)**	**.35 (.28, .41)**	**.31 (.29, .34)**	**.50**	**1.0**

*Note*. Full *r*_*A*_: full sex-limitation model allowing for quantitative sex differences and qualitative genetic sex differences; Full *r*_*C*_: full sex-limitation model allowing for quantitative sex differences and qualitative shared environmental sex differences; Common: common effects model allowing for quantitative sex differences; Scalar: scalar model; AIC: Akaike's information criterion; *A*, *C*, and *E*: the proportion of variance in depressive symptoms explained by additive genetic, shared environmental, and nonshared environmental influences, respectively; *r*_*A*_: the genetic correlation among opposite-sex twin pairs; *r*_*C*_: the shared environmental correlation among opposite-sex twin pairs; 95% confidence intervals are shown in brackets.

## References

[1] Hankin B. L., Abramson L. Y., Moffitt T. E., Angell K. E., Silva P. A., McGee R. (1998). Development of depression from preadolescence to young adulthood: emerging gender differences in a 10-year longitudinal study. *Journal of Abnormal Psychology*.

[2] Nolen-Hoeksema S., Girgus J. S. (1994). The emergence of gender differences in depression during adolescence. *Psychological Bulletin*.

[3] Ge X., Conger R. D., Elder G. H. (2001). Pubertal transition, stressful life events, and the emergence of gender differences in adolescent depressive symptoms. *Developmental Psychology*.

[4] Ge X., Lorenz F. O., Conger R. D., Elder G. H., Simons R. L. (1994). Trajectories of stressful life events and depressive symptoms during adolescence. *Developmental Psychology*.

[5] Jane Costello E., Erkanli A., Angold A. (2006). Is there an epidemic of child or adolescent depression?. *Journal of Child Psychology and Psychiatry and Allied Disciplines*.

[6] Hankin B. L., Mermelstein R., Roesch L. (2007). Sex differences in adolescent depression: stress exposure and reactivity models. *Child Development*.

[7] Hyde J. S., Mezulis A. H., Abramson L. Y. (2008). The ABCs of depression: integrating affective, biological, and cognitive models to explain the emergence of the gender difference in depression. *Psychological Review*.

[8] Frani S., Middeldorp C. M., Dolan C. V., Ligthart L., Boomsma D. I. (2010). Childhood and adolescent anxiety and depression: beyond heritability. *Journal of the American Academy of Child and Adolescent Psychiatry*.

[9] Rice F., Harold G. T., Thapar A. (2002). Assessing the effects of age, sex and shared environment on the genetic aetiology of depression in childhood and adolescence. *Journal of Child Psychology and Psychiatry*.

[10] Eley T. C., Stevenson J. (1999). Exploring the covariation between anxiety and depression symptoms: a genetic analysis of the effects of age and sex. *Journal of Child Psychology and Psychiatry*.

[11] Jacobson K. C., Rowe D. C. (1999). Genetic and environmental influences on the relationships between family connectedness, school connectedness, and adolescent depressed mood: sex differences. *Developmental Psychology*.

[12] Cho H., Guo G., Iritani B. J., Hallfors D. D. (2006). Genetic contribution to suicidal behaviors and associated risk factors among adolescents in the U.S.. *Prevention Science*.

[13] McCaffery J. M., Papandonatos G. D., Stanton C., Lloyd-Richardson E. E., Niaura R. (2008). Depressive symptoms and cigarette smoking in twins from the national longitudinal study of adolescent health. *Health Psychology*.

[14] Scourfield J., Rice F., Thapar A., Harold G. T., Martin N., McGuffin P. (2003). Depressive symptoms in children and adolescents: changing aetiological influences with development. *Journal of Child Psychology and Psychiatry and Allied Disciplines*.

[15] Eaves L. J., Silberg J. L., Meyer J. M. (1997). Genetics and developmental psychopathology: 2. The main effects of genes and environment on behavioral problems in the Virginia Twin Study of Adolescent Behavioral Development. *Journal of Child Psychology and Psychiatry and Allied Disciplines*.

[16] Happonen M., Pulkkinen L., Kaprio J., Van der Meere J., Viken R. J., Rose R. J. (2002). The heritability of depressive symptoms: multiple informants and multiple measures. *Journal of Child Psychology and Psychiatry and Allied Disciplines*.

[17] Lau J. Y. F., Eley T. C. (2006). Changes in genetic and environmental influences on depressive symptoms across adolescence and young adulthood. *The British Journal of Psychiatry*.

[18] Hunt M. (1997). *How Science Takes Stock*.

[19] Rosenthal R., DiMatteo M. R. (2001). Meta-analysis: recent developments in quantitative methods for literature reviews. *Annual Review of Psychology*.

[20] Burt S. A. (2009). Rethinking environmental contributions to child and adolescent psychopathology: a meta-analysis of shared environmental influences. *Psychological Bulletin*.

[21] Neale M., Boker S., Xie G., Maes H. (2003). *Mx: Statistical Modeling*.

[22] Rice F., Harold G., Thapar A. (2002). The genetic aetiology of childhood depression: a review. *Journal of Child Psychology and Psychiatry*.

[23] Davies P. T., Windle M. (1997). Gender-specific pathways between maternal depressive symptoms, family discord, and adolescent adjustment. *Developmental Psychology*.

[24] Crawford T. N., Cohen P., Midlarsky E., Brook J. S. (2001). Internalizing symptoms in adolescents: Gender differences in vulnerability to parental distress and discord. *Journal of Research on Adolescence*.

[25] Boyle M. H., Pickles A. (1997). Maternal depressive symptoms and ratings of emotional disorder symptoms in children and adolescents. *Journal of Child Psychology and Psychiatry*.

[26] Fergusson D. M., Horwood L. J., Lynskey M. T. (1995). Maternal depressive symptoms and depressive symptoms in adolescents. *Journal of Child Psychology and Psychiatry*.

[27] Bierut L. J., Heath A. C., Bucholz K. K. (1999). Major depressive disorder in a community-based twin sample: are there different genetic and environmental contributions for men and women?. *Archives of General Psychiatry*.

[28] Kendler K. S., Gardner C. O., Neale M. C., Prescott C. A. (2001). Genetic risk factors for major depression in men and women: similar or different heritabilities and same or partly distinct genes?. *Psychological Medicine*.

[29] Kendler K. S., Gatz M., Gardner C. O., Pedersen N. L. (2006). A Swedish national twin study of lifetime major depression. *The American Journal of Psychiatry*.

[30] Jansson M., Gatz M., Berg S. (2004). Gender differences in heritability of depressive symptoms in the elderly. *Psychological Medicine*.

[31] Johnson W., McGue M., Gaist D., Vaupel J. W., Christensen K. (2002). Frequency and heritability of depression symptomatology in the second half of life: evidence from Danish twins over 45. *Psychological Medicine*.

[32] Lamb D. J., Middeldorp C. M., van Beijsterveldt C. E. M. (2010). Heritability of anxious-depressive and withdrawn behavior: age-related changes during adolescence. *Journal of the American Academy of Child & Adolescent Psychiatry*.

[33] Vink J. M., Bartels M., van Beijsterveldt T. C. E. M. (2012). Sex differences in genetic architecture of complex phenotypes?. *PLoS ONE*.

[34] Hur Y.-M., Craig J. M. (2013). Twin registries worldwide: an important resource for scientific research. *Twin Research and Human Genetics*.

